# Niche Differentiation of Sulfate- and Iron-Dependent Anaerobic Methane Oxidation and Methylotrophic Methanogenesis in Deep Sea Methane Seeps

**DOI:** 10.3389/fmicb.2020.01409

**Published:** 2020-07-08

**Authors:** Haizhou Li, Qunhui Yang, Huaiyang Zhou

**Affiliations:** State Key Laboratory of Marine Geology, Tongji University, Shanghai, China

**Keywords:** methane seeps, South China Sea, anaerobic oxidation of methane, methylotrophic methanogenesis, sulfate reduction, iron reduction

## Abstract

Methane seeps are widespread seafloor ecosystems shaped by complex physicochemical-biological interactions over geological timescales, and seep microbiomes play a vital role in global biogeochemical cycling of key elements on Earth. However, the mechanisms underlying the coexistence of methane-cycling microbial communities remain largely elusive. Here, high-resolution sediment incubation experiments revealed a cryptic methane cycle in the South China Sea (SCS) methane seep ecosystem, showing the coexistence of sulfate (SO_4_^2–^)- or iron (Fe)-dependent anaerobic oxidation of methane (AOM) and methylotrophic methanogenesis. This previously unrecognized methane cycling is not discernible from geochemical profiles due to high net methane consumption. High-throughput sequencing and Catalyzed Reporter Deposition-Fluorescence in situ Hybridization (CARD-FISH) results suggested that anaerobic methane-oxidizing archaea (ANME)-2 and -3 coupled to sulfate-reducing bacteria (SRB) carried out SO_4_^2–^-AOM, and alternative ANME-2 and -3 solely or coupled to iron-reducing bacteria (IRB) might participate in Fe-AOM in sulfate-depleted environments. This finding suggested that ANME could alter AOM metabolic pathways according to geochemical changes. Furthermore, the majority of methylotrophic methanogens belonged to *Methanimicrococcus*, and hydrogenotrophic and acetoclastic methanogens were likely inhibited by sulfate or iron respiration. Fe-AOM and methylotrophic methanogenesis are overlooked potential sources and sinks of methane in methane seep ecosystems, thus influencing methane budgets and even the global carbon budget in the ocean.

## Introduction

Methane seeps are methane-dependent chemosynthetic ecosystems (Paull et al., 1984) that occur widely in the marine environment, and are considered some of the richest benthic ecosystems on the seabed (Valentine, 2011). The microbially mediated methane cycle dominates methane seeps (Boetius et al., 2000; Knittel and Boetius, 2009) and has an important impact on the global carbon cycle (Boetius and Wenzhöfer, 2013; Offre et al., 2013). Methane is produced from the degradation of organic matter by methanogens in deep sediment (Reeburgh, 2007). The produced methane continuously seeps or erupts from the sedimentary subsurface to the seabed in the form of methane-rich fluids, and more than 90% of the methane is consumed by anaerobic methane-oxidizing archaea (Valentine, 2011), forming an efficient methane biofilter that prevents its diffusion into the seawater.

Previous studies believed that in the ocean, the majority of methane is oxidized anaerobically by anaerobic methane-oxidizing archaea (ANME) coupled with sulfate reduction (Boetius et al., 2000). However, various chemical compounds are thermodynamically more favorable electron acceptors than sulfate for catalyzing anaerobic methane oxidation, such as nitrite (Raghoebarsing et al., 2006), nitrate (Haroon et al., 2013), ferric iron, and manganese (Ettwig et al., 2016; Cai et al., 2018). For instance, biogeochemical profiling evidences indicated the widespread presence of Fe-anaerobic oxidation of methane (AOM) in the ocean, such as in Argentine Basin (Riedinger et al., 2014), Alaskan Beaufort Sea (Treude et al., 2014), North Sea Helgoland mud (Oni et al., 2015), Baltic Sea (Egger et al., 2017), and Mediterranean Sea (Vigderovich et al., 2019). Targeted enrichment with ferrihydrite provides strong evidence for AOM coupled with iron reduction, and ANME-1, *Methanococcoides*/ANME-3 (Beal et al., 2009), ANME-2a and -2c (Scheller et al., 2016), ANME-2d (*Methanoperedens nitroreducens*) (Ettwig et al., 2016; Shen et al., 2019), Candidatus *Methanoperedens ferrireducens* (Cai et al., 2018), and *Methanosarcina acetivorans* (Yan et al., 2018) might be involved in Fe-AOM. However, the Fe-AOM microorganism physiology and its contribution to the methane consumption remain poorly understood in deep sea (Scheller et al., 2016; He et al., 2018). It is still possible that other unknown microorganisms can perform metal-AOM. For instance, Bar-Or et al. (2017) highlighted the essential role of methanogens and methanotrophic bacteria in the process of Fe-AOM.

In addition, in methane seep ecosystems, acetoclastic and hydrogenotrophic methanogenesis are often considered the dominant methanogenic processes below the Sulfate-methane transition zone (SMTZ) (Zhe et al., 2018). Thermodynamic laws indicate that sulfate-reducing bacteria (SRB) and iron-reducing bacteria (IRB) outcompete methanogens for hydrogen and acetate substrates, thus inhibiting the acetoclastic/hydrogenotrophic methanogenesis pathways (Reeburgh, 2007; Reiche et al., 2010; Zhou et al., 2014; Zhuang et al., 2018). However, the co-occurrence of sulfate reduction, AOM, and methanogenesis has been demonstrated in marine sediments (Sivan et al., 2014). This is also partly supported by the coexistence of ANME and methanogenic archaea in Sonora Margin shallow sediments (0–20 cm) (Vigneron et al., 2015), Peruvian Margin sulfate-reducing zone (0–25 cm) (Maltby et al., 2016), Mediterranean Sea shallow sediment (0–20 cm) (Sela-Adler et al., 2017), and Aarhus Bay surface sediment (Xiao et al., 2017, 2018).

Then, it is becoming clear that methylotrophic methanogenesis plays a major role in marine sediments, especially surficial sediments (Zhuang et al., 2016, 2018; Xiao et al., 2017). Methylotrophic methanogenesis using non-competitive substrates such as (methanol, methylamines, or methyl sulfide and so on) could co-occur with AOM and sulfate reduction (Vigneron et al., 2015; Zhuang et al., 2016). In the presence of sulfate and iron oxides, methanogens may circumvent competition by utilizing ‘non-competitive’ methylated substrates. These non-competitive substrates are ubiquitous in the marine environment, and originate from the degradation of substances such as betaine, choline, lignins, pectin, and creatine, or from the bacterial reduction of trimethylamine oxide (Oremland et al., 1982). Hence, methylotrophic methanogenesis has been suggested to occur in all major oceans (Valentine, 2011; Chronopoulou et al., 2017). However, in methane seep ecosystems, this process is not easily discernible from geochemical profiles due to the overall high net methane consumption in methane seeps. Thus, research on methylotrophic methanogenesis in methane seep is relatively rare, and the contribution of methylated substrates to methanogenesis processes remains elusive (Chronopoulou et al., 2017).

The northern continental slope of the South China Sea (SCS) has various sites of methane seepage, covering a wide range of water depths (200–3000 m). The Jiaolong and Haima methane seeps are the two still-active seep sites in SCS. Previous studies on the seeps in SCS have led to many significant advancements including new insight into the methane seep structure (Liu et al., 2018), mineral carbon/oxygen/sulfur isotopes (Feng et al., 2018), macrobenthos (Dong and Li, 2015; Gong et al., 2015), and microbial lipid biomarkers (Guan et al., 2016, 2018). In addition, the microbial distribution and diversity in SCS methane seeps were also investigated (Jiang et al., 2007; Zhang et al., 2012), and a 16S rRNA gene-based survey indicated the presence of SRB and ANME. However, the biogeochemical evidences and potential activity of methane-cycling microorganisms and their niche differentiation patterns are largely unknown.

In this study, the Jiaolong methane seep was chosen as our research object. In 2018, a remotely operated underwater vehicle, The Remotely Operated Platform for Ocean Science (ROPOS), found a new methane seepage site in this area. Combining high-throughput sequencing, CARD-FISH, and enrichment culture methods with pore water biogeochemistry, we investigated the microbially driven metabolic processes of methane production and consumption. The coexistence of sulfate- or iron-dependent AOM and methylotrophic methanogenesis was found, and the potential rates of these processes were assessed. These results are of great importance to the understanding of the biogeochemical processes in global methane seep ecosystems.

## Materials and Methods

### Collection of Sediment Samples and Geochemical Analysis

**FIGURE 1:**
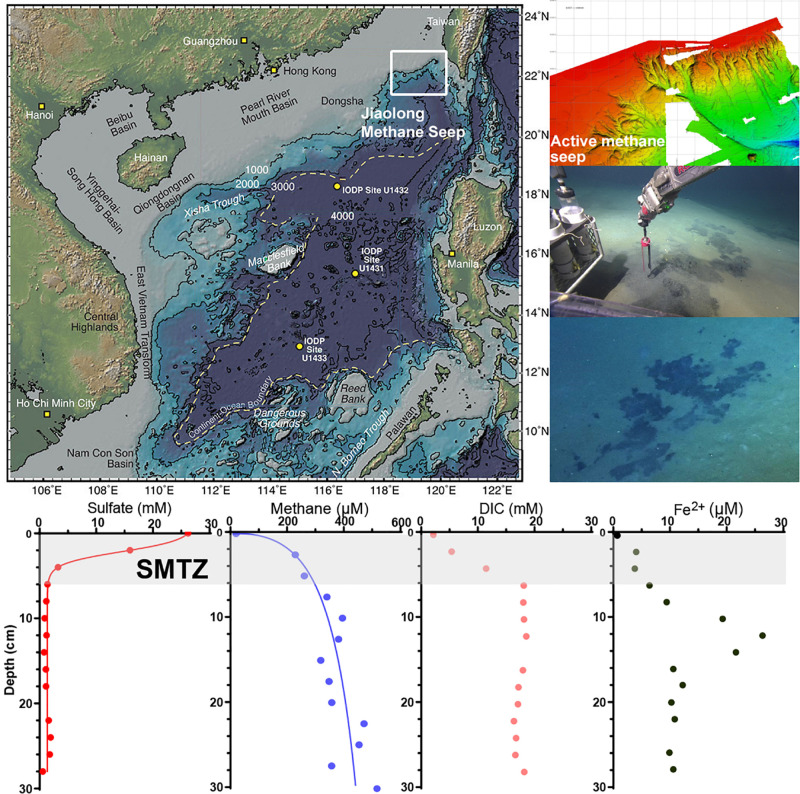
The location of Jiaolong active methane seep and geochemistry profiles. The map is modified from Expedition 349 Scientists (2014).

During the R/V Tan Kah Kee 1083 expedition (April–May 2018) in the northern SCS, a remotely operated underwater vehicle (ROPOS, Canadian Scientific Submersible Facility) was used to search for new undisturbed active methane seeps at a water depth of 900–1,200 m. A new active methane seep was found and named Jiaolong F3 site (position 22°6.9678′N, 119°17.0841′E, 1162.53 m). Two push cores with a length of 32 cm were retrieved beneath the black microbial mats by ROPOS (Figure 1). One push core was used for shipboard biogeochemical analyses, and the other push core was stored at 4°C until it was taken back to the laboratory for enrichment experiments.

Before sampling, holes were drilled at both sides of the biogeochemistry push cores to collect porewater and methane gas. Holes in one side were drilled at 2 cm intervals, in the other at 2.5 cm, and sealed with diffusion-tight tape. Porewater was extracted using Rhizons (Rhizosphere Research Products, Wageningen, Netherlands) at 2 cm intervals (Seeberg-Elverfeldt et al., 2005). Cut-off syringes were used to sample sediment at a depth interval of 2.5 cm for methane analysis. The push core was then sectioned into 2 cm thick slices and frozen at −80°C for nucleic acid extraction and CARD-FISH analysis. The collected pore water was divided into aliquots for analysis of sulfate, dissolved inorganic carbon (DIC), and dissolved iron (Fe^2+^).

The pore water sample used for analysis of DIC was quickly transferred into glass vials (2 ml), leaving no headspace for concentration measurements and stored at 4°C. In the laboratory, pore water was measured using a CN analyzer multi N\C 3100 (Analytik Jena AG, Jena, Germany) (Oda et al., 2019). For analysis of the sulfate concentration, 1 ml of pore water was transferred into a vial containing 0.5 ml of ZnAc (5%), which was shaken well and stored at −20°C. In the laboratory, sulfate was measured using a Dionex ICS-1500 Ion Chromatograph and an IonPac AS23 column (eluent: 4.5 mM Na_2_CO_3_/0.8 mM NaHCO_3_, flow: 1 ml/min) (Thermo Fisher Scientific, Sunnyvale, CA, United States). For measurement of dissolved iron, 1 ml of pore water was acidified with ultrapure HCl and assessed using a 797 VA Computrace as previously described (Metrohm, Herisau, Switzerland).

For *ex situ* measurement of the methane concentration, 3 ml of wet sediment sample was removed from the push core at an interval of 2.5 cm with a cut-off syringe and extruded into a vial (20 ml) containing 6 ml of sodium hydroxide (2.5% w/w). The vial was closed immediately with a butyl rubber stopper, sealed with an aluminum crimp, and stored upside down at 4°C until measurement by gas chromatography (Treude et al., 2005). Before measurement, the sealed bottle was vigorously shaken and headspace methane was determined using gas chromatography-flame ionization detector (Agilent, Santa Clara, CA, United States).

### High-Throughput Sequencing

Environmental DNA was extracted using a modified SDS-based extraction method (Zhou et al., 1996). The final DNA concentration was determined by a NanoDrop 2000 (Thermo Scientific, Waltham, MA, United States), and DNA quality was checked by 1% agarose gel electrophoresis. The sequencing steps were conducted by Majorbio Bio-Pharm Technology Co., Ltd. (Shanghai, China). The bacterial primers were 338F and 806R (Xu et al., 2016), targeting the 16S rDNA V3-V4 region. The archaeal primers were 524F10extF and Arch958R modR (Liu C. et al., 2016), targeting the 16S rDNA V4-V5 region. The PCR protocol was performed according to previously described methods (Liu C. et al., 2016; Xu et al., 2016). The PCR products were extracted from a 2% agarose gel and further purified using an AxyPrep DNA Gel Extraction Kit (Axygen Biosciences, United States) and quantified using Quanti Fluor^TM^-ST (Promega, United States). Purified amplicons were pooled in equimolar amounts and paired-end sequenced (2 × 300) on an Illumina MiSeq platform (Illumina, San Diego, CA, United States). Raw fastq files were demultiplexed, quality filtered by Trimmomatic, and merged by FLASH. Operational taxonomic units (OTUs) were clustered with a 97% similarity cutoff using UPARSE (version7.1), and chimeric sequences were identified and removed using UCHIME. Taxonomy assignment was performed using the SILVA 16S rRNA database (version 132). The distance-based maximum likelihood was used for phylogenetic analysis. Bootstrap analysis was performed using 1000 replications. Chao1 and Shannon–Weaver diversity indices and rarefaction curves were calculated by MOTHUR. Principal coordinates analysis (PCoA) was computed using PAST. Sequencing data are stored on the NCBI Sequence Read Archive (PRJNA574743, PRJNA574745).

### Catalyzed Reporter Deposition-Fluorescence *in situ* Hybridization (CARD-FISH)

The CARD-FISH protocol was based on a previous study (Pernthaler et al., 2002). The probes-label peroxidase is shown in Table 1. Sediment (0.5 g) was fixed with 4% paraformaldehyde for 24 h at room temperature. The fixed sediments were washed three times by centrifugation (8,000 × *g* for 10 min) using PBS at 4°C and stored in ethanol/PBS buffer (1:1) at −20°C for further processing. After that, 100 μl of fixed sediments were diluted with 900 μl ethanol/PBS buffer (1:1) and dispersed using ultrasound. Then, 20 μl of dispersed sediments were diluted in 20 ml of Milli Q filtered water. The suspension sediments were filtered on polycarbonate filters, and 0.1% low melting point agarose was dripped onto the filters and dried at 46°C in an incubator. The microbes were permeabilized using 15 μg/ml proteinase K. Then, 3% H_2_O_2_ was used to inactivate the endogenous peroxidases.

**TABLE 1:** Oligonucleotide probes.

Oligonucleotides	Specificity	FA(%)	Nucleotide sequence 5′-3′	References
EUB338	Bacteria	35	GCW GCC WCC CGT AGG WGT	Eickhorst and Tippkötter, 2008
Arch915	Archaea	35	GTG CTC CCC CGC CAA TTC CT	Eickhorst and Tippkötter, 2008
ANME-1-350	ANME-1	40	AGT TTT CGC GCC TGA TGC	Boetius et al., 2000
ANME-2-538	ANME-2	35	GGC TAC CAC TCG GGC CGC	Nauhaus et al., 2005
ANME-3-1249	ANME-3	35	TCG GAG TAG GGA CCC ATT	Krüger et al., 2005
DSS-658	Desulfosarcina/Desulfococcus	50	TCC ACT TCC CTC TCC CAT	Manz et al., 1992
SEEP-1a-1441	SEEP-SRB1	45	CCC CTT GCG GGT TGG TCC	Schreiber et al., 2010
SEEP-2-658	SEEP-SRB2	45	TCC ACT TCC CTC TCC GGT	Kleindienst et al., 2012
SEEP3-652	SEEP-SRB3	50	TAC CCC CTC TGG TAC TCA	Orcutt et al., 2010
SEEP4-583*	SEEP-SRB4	20	CTG ACA TAA CAR ACC ACC	Orcutt et al., 2010
NON338	Nonsense probes	35	ACT CCT ACG GGA GGC AGC	Wallner et al., 1993

For hybridization, filters were placed in a tube and mixed with 500 μl hybridization solution [10% dextran sulfate, 2% blocking reagent (Roche, Germany), 0.1% (w/v) sodium dodecyl sulfate, 20 mM Tris–HCl [pH 8.0], 0.9 M NaCl and formamide], and 1 μl of probe working solution (final concentration, 0.028 μM) (Eickhorst and Tippkötter, 2008). Microorganisms were hybridized for at least 60 min on a rotor at 46°C; then, the filters were washed twice using washing solution (Eickhorst and Tippkötter, 2008) (0.01% SDS, 5 mM EDTA [pH 8.0], 20 mM Tris–HCl [pH 8.0] and 3 mM NaCl) at 48°C for 20 min. After washing, filters were mixed with 1000 μl of amplification solution (0.0015% H_2_O_2_, 1 × PBS [pH7.4], 0.1% (w/v) blocking reagent) and 1 μl of Alexa488 labeled tyramides (Life Technologies^TM^, Thermo Fisher, United States). The probes were incubated at 46°C in amplification solution for at least 30 min in the dark.

For second hybridizations, the first probe-label peroxidase was inactivated by incubating the filter sections in 0.01 M HCl for 10 min at room temperature and washing the sections with 50 ml of Milli Q water. Then, the CARD-FISH protocol was repeated two times with the same filter sections by using different probes. The second hybridization used Alexa 647 labeled tyramides (Life Technologies^TM^, Thermo Fisher, United States).

Finally, all microorganisms were stained using 4′,6-diamidino-2-phenylindole (DAPI) and mounted with ProLong Gold Antifade reagent (Life Technologies, Carlsbad, CA, United States). Cell counting was performed using ImageJ.

### Incubation Experiments for Methane Metabolic Activity

The push core (30 cm length) stored at 4°C was processed for microbial AOM and methanogenesis activity. We determined which substrates were available for AOM and methanogenesis in the Jiaolong methane seep. Push cores were positioned vertically, and the sediment was pushed out of the liner using a plastic plunger. From the center of the push core, samples were taken and incubated for determination of AOM and methanogenesis activity.

Methods for determining potential rates of AOM have been published previously (Segarra et al., 2015). For measurements of the potential anaerobic methane oxidation rate and mechanism, 5 g of sediment from a different depth was added to 120 ml serum bottles containing 50 ml of artificial mineral medium, with different treatments (see Table 2): (1) 5 mM sulfate, (2) 10 mM Ferrihydrite + 20 mM molybdate, (3) 5 mM Nitrite + 20 mM molybdate + 20 mM 2-bromoethanosulfonate (BES), (4) 10 mM Nitrite + 20 mM molybdate + 20 mM BES, (5) 20 mM molybdate + 20 mM BES, (6) Without any additions. Molybdate as inhibitor for sulfate reduction and BES as inhibitor for methanogenesis (Chidthaisong and Conrad, 2000; Nauhaus et al., 2005) were added. Another reason for BES not inhibiting the ANME archaea (or only partially inhibiting them) could be the formation of syntrophic clusters, especially by ANME-2 archaea, that are not fully permeated by BES (Dekas et al., 2009; Haroon et al., 2013). Negative controls were incubated in parallel for each process within each depth interval so that live samples could be corrected for this ‘background’ activity. Ferrihydrite was synthesized according to protocols described previously (Cai et al., 2018). The serum bottles were sealed with butyl rubber stoppers, crimp-capped, degassed with N_2_, and reduced with Na_2_S ⋅ 9H_2_O (0.5 g/L) and L-cysteine (0.5 g/L). The bottles were filled with 10% methane and incubated for 6 months at 4°C in the dark (Beal et al., 2009). Then, the accumulation of DIC was measured by a C/N elemental analyzer (N/C^®^ 3100, Analytik Jena AG, Germany) as a response to AOM activity. Initial DIC concentrations in the medium and those at time-points DIC were determined.

**TABLE 2:** Incubation experiments.

Treatments	Depth (cm)							
	0–2	4–6	8–10	12–14	16–18	20–22	24–26	28–30
**AOM (nmol g^–1^d^–1^)**								
(1) Without any addition	0.257	1.257	2.047	1.258	0.625	0.120	0.150	0.101
(2) Sulfate (5 mM)	10.650	401.204	902.248	801.574	780.228	750.144	600.688	580.890
Net SO_4_^2–^-AOM	10.393	399.947	900.201	800.316	779.603	750.024	600.538	580.789
(3) BES + Molybdate	–	–	0.032	0.025	0.031	0.035	0.026	0.035
(4) BES + Molybdate + Ferrihydrite (10 mM)	–	–	30.034	38.019	20.433	35.055	35.023	30.034
Net Fe-AOM	–	–	30.002	37.994	20.402	35.020	34.997	29.999
(5) BES + Molybdate + Nitrite (5 mM)	–	–	0.031	0.025	0.033	0.033	0.025	0.031
Net NO_2_^–^-AOM	–	–	0	0	0.002	0	0	0
(6) BES + Molybdate + Nitrate (10 mM)	–	–	0.030	0.020	0.029	0.031	0.024	0.037
Net NO_3_^–^-AOM			0	0	0	0	0	0.002
**Methanogenesis (nmol g^–1^d^–1^)**								
(1) Molybdate	–	0.023	0.083	0.082	0.172	0.132	0.122	0.104
(2) Molybdate + Methanol (20 mM)	–	0.042	2.082	3.080	6.021	5.933	5.424	5.105
Net Methylotrophic methanogenesis	–	0.019	1.999	2.998	5.849	5.801	5.302	5.001
(3) Molybdate + H_2_/CO_2_ (80/20%)	–	0.012	0.083	0.091	0.164	0.133	0.123	0.115
Net hydrogenotrophic methanogenesis	–	0	0	0.009	0	0.001	0.001	0.011
(4) Molybdate + Acetate (20 mM)	–	0.020	0.075	0.084	0.173	0.152	0.110	0.090
Net acetoclastic methanogenesis	–	0	0	0.002	0.001	0.020	0	0

*BES (20 mM), 2-bromoethanosulfonate, methanogenesis inhibitor; Molybdate (20 mM): inhibitor of SO_4_^2–^-AOM. Rates were collected in duplicate; –, rates not detectable.*

Furthermore, the potential methane production rate and mechanism were assessed (Buckley et al., 2008; Xiao et al., 2018). Five grams of different depth sediments were added to 120 ml serum bottles containing 50 ml of artificial mineral medium with different treatments: (1) molybdate (20 mM), (2) hydrogen under H_2_/CO_2_ 80/20 atmosphere + molybdate (20 mM), (3) acetate (20 mM) + molybdate (20 mM), and (4) methanol (20 mM) + molybdate (20 mM). Molybdate was used as an enzymatic inhibitor for sulfate reduction (Oremland and Capone, 1988). Samples with molybdate additions served as negative controls for sulfate-dependent methane oxidation. NaHCO_3_ was added to a concentration of 10 mM to make a buffer of CO_2_-HCO_3_^–^CO_3_^2–^ to maintain the pH of the medium around neutral. Measurements indicated that the pH was in the range of 7.0–7.2 at the start of the experiment. The serum bottles were sealed with butyl rubber stoppers, crimp-capped, degassed with N_2_, and reduced with Na_2_S ⋅ 9H_2_0 (0.5 g/L) and L-cysteine (0.5 g/L). Then, the sediments were incubated for 6 months at 4°C in the dark. Methane production was measured by sampling the headspace of the serum vials by syringe and analyzing with GC-FID (Agilent, United States), using manual injection of 100 μl of headspace gas (Buckley et al., 2008). The needles used for sampling the serum vial headspace were flushed with N_2_ prior to sampling to prevent oxygen intrusion into the serum vials.

The artificial mineral medium contained (per 1 L): 23 g NaCl, 0.0136 g KH_2_PO_4_, 0.0535 g NH_4_Cl, 0.0147 g CaCl_2_ ⋅ 2H_2_O, 0.0204 g MgCl_2_ ⋅ 6H_2_O, 1 ml trace element solution (Könneke et al., 2005), and 1 ml vitamin solution (Könneke et al., 2005).

## Results

### Geochemistry Profiles

**FIGURE 2:**
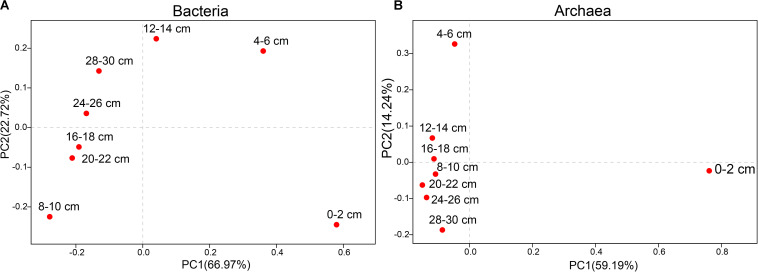
Principal coordinates analysis (PCoA) of different sediments at the genus level. **(A)** Bacteria. **(B)** Archaea.

The geochemistry results showed that the concentrations of sulfate, methane, and DIC changed rapidly in the depth range of 0–6 cm (Figure 2). The concentration of sulfate decreased from nearly 26.2 mM to 0.43 μM, that of methane increased from 30 μM to 320 μM, and that of DIC increased from 2.20 to 18.34 mM. The rapid decrease in sulfate concentration as well as increase in DIC and methane concentrations indicate that the SMTZ in Jiaolong methane seep area is shallow, within 6 cm below the sediment-water interface. The concentration of pore water ferrous iron was the lowest in the surface sediment (2.6 μM), peaked at a depth of 12 cm (26.6 μM), and then decreased to 10 μM. The highest concentration of ferrous iron appeared below the SMTZ, where sulfate has been exhausted.

### Microbial Diversity

The vertical distribution of the microbial community was investigated in sediments from different depths (Table 3 and Supplementary Figure S1). In total, 848,371 high-quality sequences were generated from the samples (bacteria = 404662 sequences, archaea = 443709 sequences). At the 97% sequence identity level, 114480 OTUs were identified (bacteria = 57767 OTUs, archaea = 56713 OTUs). We found obvious differences in microbial structures among different sediment depths, and archaeal diversity was lower than bacterial diversity.

**TABLE 3:** Microbial diversity index.

Depth	Ace	Chao1	Coverage	Shannon	Simpson	Sobs
**Bacteria**						
0–2 cm	1180	982	0.9952	2.08	0.4037	669
4–6 cm	2196	2234	0.9948	6.19	0.0056	2016
8–10 cm	2010	2026	0.9892	5.02	0.0391	1695
12–14 cm	2153	2178	0.9941	6.11	0.0104	2043
16–18 cm	2215	2202	0.9927	5.80	0.0156	2038
20–22 cm	2205	2253	0.9925	5.75	0.0219	2042
24–26 cm	2182	2200	0.9944	5.79	0.0140	2049
28–30 cm	1744	1779	0.9907	5.94	0.0093	1607
**Archaea**						
0–2 cm	86	84	0.9997	1.23	0.5085	82
4–6 cm	174	180	0.9988	1.89	0.2790	143
8–10 cm	207	214	0.9990	1.95	0.6906	159
12–14 cm	276	282	0.9996	2.70	0.1750	267
16–18 cm	259	257	0.9994	1.88	0.3739	247
20–22 cm	271	288	0.9991	1.92	0.3812	251
24–26 cm	252	251	0.9995	2.02	0.3174	242
28–30 cm	257	257	0.9997	2.19	0.2705	254

**FIGURE 3:**
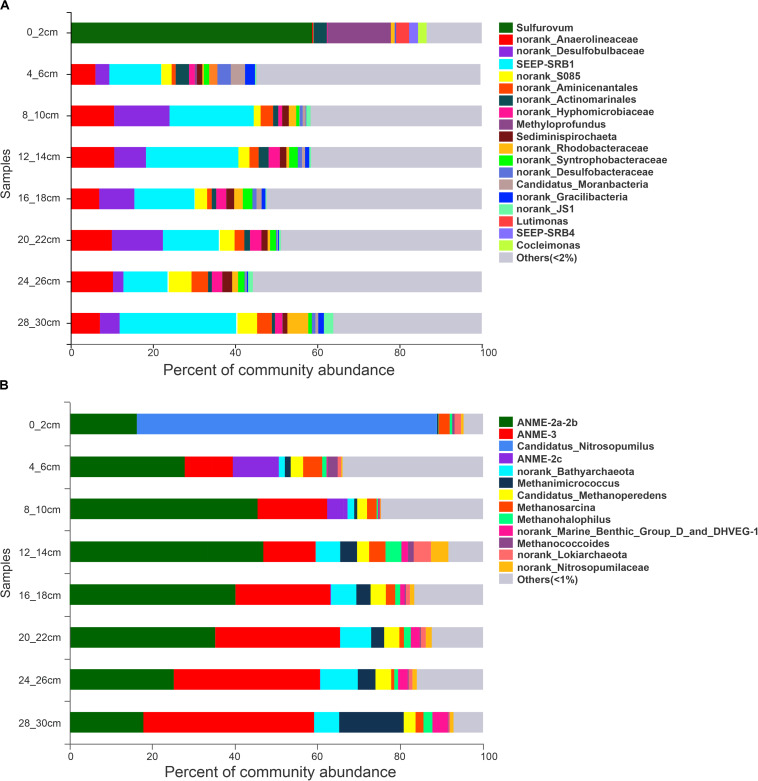
16S rRNA gene sequence abundance at the genus level obtained from sediments at different depths. **(A)** bacteria. **(B)** archaea.

**FIGURE 4:**
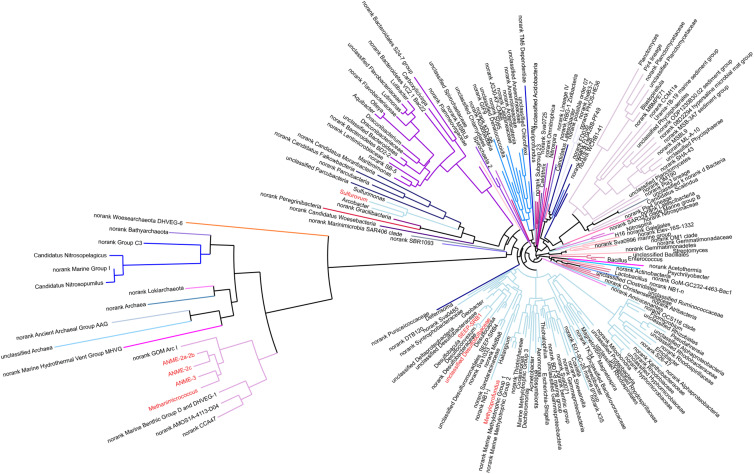
Phylogenetic relationships of 200 representative microbial sequences. Colored lines represent each phylum. The red-colored genus indicates dominant microbes, such as ANME-2a-2b, ANME-2c, ANME-3, *Methanimicrococcus*, *Sulfurovum*, SEEP-SRB1, *Methyloprofundus* etc.

The PCoA of bacteria from all samples showed differences between surface sediments and deeper sediments (Figure 2). The species diversity was the lowest in surface sediments (Shannon: 2.08), and the highest species diversity was found at the depths of 4–6 cm and 12–14 cm (Shannon: 6.19 and 6.11, respectively). In surface sediments, most of the bacterial sequences were clustered in *Sulfurovum* (60.0179%), *Methyloprofundus* (11.1006%), and *Sulfurimonas* (3%). In the sediments at depths of 4–28 cm, the dominant groups belonged to SRB, including SEEP-SRB1 (12–28%), and Unclassified Desulfobulbaceae (8–20%) (Figures 3, 4). The relative abundance of SEEP-SRB4 was relatively low (0.07–0.4%). In addition, different kinds of IRB were detected including *Shewanella* (0.01–0.07%), *Pseudomonas* (0.01–0.1%), *Desulfuromonas* (0.11-0.92%), *Geobacter* (0.004–0.02%), and so on (Figure 4).

The PCoA of archaea from all samples showed differences between sediments at depths of 0–6 cm and deeper sediments (Figure 2). The species diversity was the lowest in surface sediments (Shannon: 1.23), and the highest species diversity was found at a depth of 12–14 cm (Shannon: 2.70). In surface sediments, the dominant sequence cluster included mainly *Nitrosopumilus* (70.65%) and ANME-2a/b (18.84%). As the depth of sediment increased, *Nitrosopumilus* rapidly disappeared, while ANME-2a/b became more abundant, peaking (44.5%) at the depth of 8-10 cm, and then decreased. ANME-3 appeared at the depth of 4–6 cm, and its abundance increased with depth. ANME-2a/b was replaced by ANME-3 at the depth of 20–22 cm. ANME-2c appeared mainly in sediments at depths of 4–6 cm (Figure 3B). Methanogenic archaea were dominated by *Methanimicrococcus* at depths of 4–30 cm, whose abundance gradually increased from 0.20% to 18.00% with depth, with the highest abundance in the deepest sediments (28–30 cm) (Figures 3, 4). Furthermore, low abundance of methanogens below 4 cm depth was also detected, such as that of norank_Bathyarchaeota (0.01–7%), *Methanosphaera* (0.0014–0.0024%), *Methanomethylovorans* (0.0014–0.0076%), *Methanosalsum* (0.0014–0.0024%), *Methanolobus* (0.0178–0.0855%), *Methanohalophilus* (0.31–3.83%), *Methanococcoides* (0.09–2.34%), *Methermicoccaceae* (0.004–0.05%), and *Methanomassiliicoccus* (0.0051–0.0136%) (Figures 3, 4).

### Relative Cell Distribution and Abundance of Microorganisms

**FIGURE 5:**
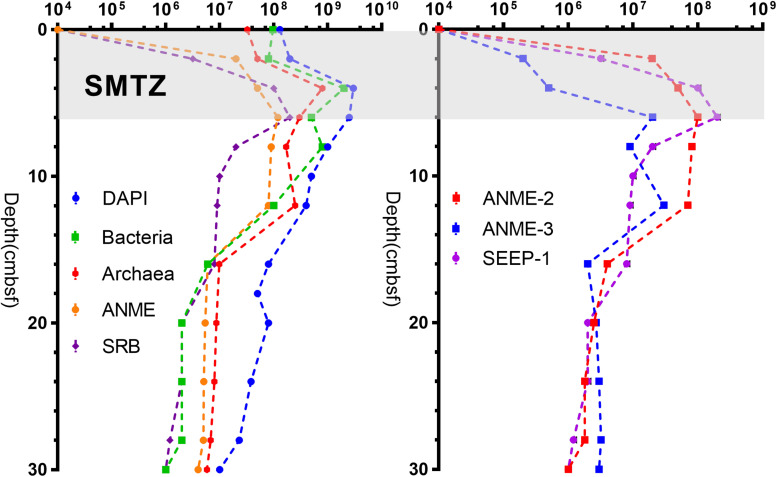
Cell number data for different sediment samples collected from the Jiaolong methane seep.

As shown in Figure 5, the distribution of bacteria, archaea, ANME, and SRB were determined by CARD-FISH. In surface sediments, the total number of cells was 1.3 × 10^8^ cells g^–1^ (3.25 × 10^7^ cells g^–1^ for archaea and 9.7 × 10^7^ cells g^–1^ for bacteria) with bacteria as the dominant type. The numbers of SRB and ANME were below the detection limit. At the depth of 2–6 cm, the number of cells increased sharply, reaching 3.0 × 10^9^ cells g^–1^ at a depth of 4 cm (2.0 × 10^9^ cells g^–1^ for bacteria and 8.0 × 10^8^ cells g^–1^ for archaea). Below 6 cm, the total number of cells decreased with increasing depth, reaching 1.0 × 10^7^ cells g^–1^ at the bottom layer (1.1 × 10^6^ cells g^–1^ for bacteria and 5.8 × 10^6^ cells g^–1^ for archaea).

SEEP-SRB1 was the dominant group among SRB, and there was a relatively low concentration of SEEP-SRB4. ANME was dominated by ANME-2 and ANME-3. ANME-1, SEEP-SRB2, and SEEP-SRB3 were not detected by CARD-FISH. The numbers of SRB and ANME were positively correlated at the depths of 0–6 cm, both increasing with depth and peaking at a depth of 6 cm (2.0 × 10^8^ cells g^–1^ for SRB and 1.4 × 10^8^ cells g^–1^ for ANME). As the depth increased, the number of SRB gradually decreased to 1.0 × 10^6^ cells g^–1^. The number of ANMEs was maintained at 9.0 × 10^7^ cells g^–1^ at depths of 6–12 cm and gradually decreased to 4.1 × 10^6^ cells g^–1^ at the bottom. Notably, there was no significant change in the abundance of ANME at depths of 6–12 cm, whereas the number of SRB decreased rapidly (Figure 5). In the surface and middle sediments, ANME-2 was dominant, while in the bottom sediments, ANME-3 was dominant (Figure 5).

**FIGURE 6:**
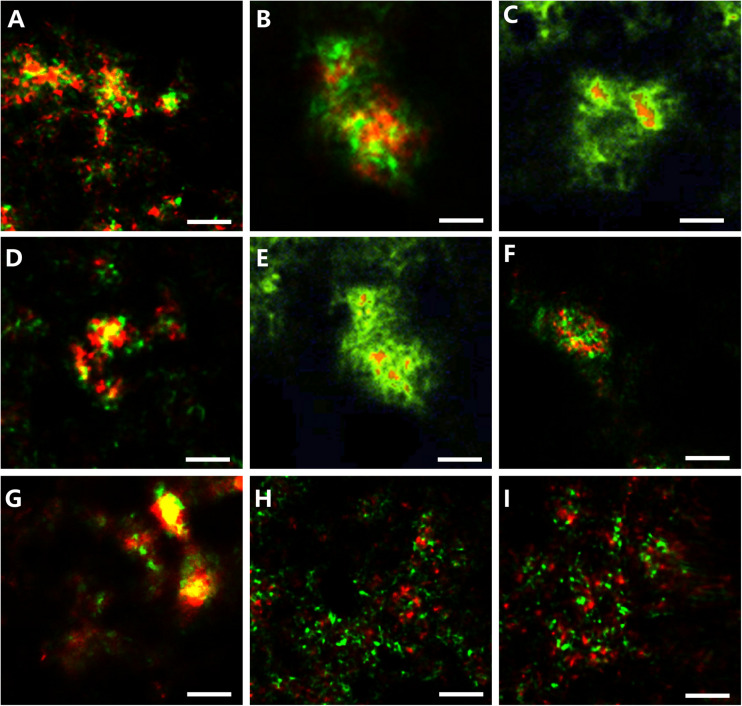
Photomicrographs of CARD-FISH stained samples. **(A)** Archaeal/bacterial aggregate labeled with ARC915 (red) and EUB338 (green) probes. **(B,C)** ANME-2 (red, ANME-2-538) and SRB1 (green, probe: SEEP-1a-1441). **(D,E)** ANME-3 (red, probe: ANME-3-1249) and SEEP-SRB1 (green, probe: SEEP1a-1441). **(F)** ANME-2 (red, probe: ANME-3-1249) and SEEP-SRB4 (green, probe: SEEP4-583). **(G)** ANME-3 (red, probe: ANME-3-1249) and SEEP-SRB4 (green, probe: SEEP4-583). **(H)** Monospecific aggregate of ANME-2 (red, probe: ANME2–538, green probe: EUB338). **(I)** Monospecific aggregate of ANME-3 (red, probe: ANME3-1249, green probe: EUB338).

Morphological studies showed that most of the SEEP-SRB and ANME formed conspicuous AOM consortia (Figure 6). The archaeal core consisted of ANME-2 and ANME-3, and the envelope consisted of SEEP-SRB1 or SEEP-SRB4. SEEP-SRB1 coupled most of ANME-2 and ANME-3, and only a small quantity of ANME-2 and ANME-3 were coupled by SEEP-SRB4. Moreover, we found SEEP-SRB and ANME single cells at different depths. Especially at depths of 10–14 cm, the numbers of ANME-2 and ANME-3 single cells were higher than those in other sediments.

### Incubation Conditions of Methane-Metabolizing Microorganisms

AOM activities was assessed as shown in Table 2. With the addition of sulfate, the net rate of SO_4_^2–^-AOM was 10.39 nmol cm^–3^ day^–1^ in surface sediments (0–2 cm), and the net rate of SO_4_^2–^-AOM increased to its maximum of 900.20 nmol cm^–3^ day^–1^ at a depth of 8–10 cm, below which the rate decreased, reaching 580.79 nmol cm^–3^ day^–1^ at a depth of 28–30 cm.

In other experiments, sulfate reduction was inhibited with the addition of molybdate (20 mM), and methanogenesis was also inhibited by 20 mM BES. When ferrihydrite was supplied, the net Fe-AOM activity appeared below the SMTZ at a depth of 8–10 cm with its rate reaching 30.00 nmol cm^–3^ day^–1^. The highest rate of Fe-AOM was 37.99 nmol cm^–3^ day^–1^ at a depth of 12–14 cm where the concentration of ferrous iron was the highest (26.60 μM). Furthermore, no obvious increase in nitrite/nitrate-dependent AOM activity had been detected. These results suggested the potential existence of Fe-AOM below the SMTZ in the Jiaolong methane seep.

Sequencing data showed the coexistence of methanogenic archaea and ANME in the sediments, suggesting the cooccurrence of methanogenesis and AOM. Hence, an enrichment experiment was carried out to test our hypothesis. All cultures were incubated at 4°C for 6 months. The data showed that there was no methanogenic potential in the surface sediments. The efflux of methane appeared at the depth of 4–6 cm, with its rate reaching 0.02 nmol cm^–3^ day^–1^. The rate peaked at 0.17 nmol cm^–3^ day^–1^ at a depth of 16–18 cm, and then decreased to 0.10 nmol cm^–3^ day^–1^ at a depth of 28–30 cm (Table 2). Furthermore, methanol, H_2_/CO_2_, and acetate were added to the sediments. The results showed that there was no obvious increase in methane production with the addition of H_2_/CO_2_ and acetate. However, stimulation of methane production was detected after methanol was added. In the sediments with a depth of 16–18 cm, the net rate of methylotrophic methanogenesis reached its maximum (5.85 nmol cm^–3^ day^–1^).

## Discussion

### Niches and Diversity of Microbes in the Jiaolong Methane Seep

We compared the archaeal and bacterial diversity of the Jiaolong methane seep with those of 23 globally distributed methane seeps, and found that the microbial richness of the Jiaolong methane seep was at a medium level (Supplementary Figure S3). However, microbial abundance data showed that the Jiaolong methane seep hosted higher biomass (5.8 × 10^6^–2.0 × 10^8^ cells g^–1^ for archaea,1.1 × 10^6^–2.0 × 10^9^ cells g^–1^ for bacteria) than other methane seeps in the SCS, such as the Haima methane seep (2.8 × 10^4^–3.4 × 10^6^ cells g^–1^ for archaea, 4.5 × 10^5^–7.4 × 10^6^ cells g^–1^ for bacteria) (Niu et al., 2017), the GMGS2 gas hydrate station (1.3 × 10^4^–2.7 × 10^6^ cells g^–1^ for archaea, 3.8 × 10^4^–1.0 × 10^7^ cells g^–1^ for bacteria) (Cui et al., 2019), and the Haiyang 4 hydrate station (with total cells of 10^5^–10^6^ cells g^–1^) (the microbial abundance was converted from 16S rDNA qPCR data) (Zhang et al., 2012).

It was found that in the Jiaolong methane seep, the main microbes were SEEP-SRB1 coupled to ANME-2 and ANME-3, and a few SEEP-SRB4 coupled to ANME-2 and ANME-3. No ANME-1, SEEP-SRB2, or SEEP-SRB3 were observed in the sediments. In the surface layer, the dominant sequence cluster belonged mainly to *Sulfurovum*, *Methyloprofundus*, *Sulfurimonas, Nitrosopumilus*, and ANME-2a/b. In contrast, in the deepest sediments, the dominant sequence cluster belonged mainly to ANME-2, ANME-3, and *Methanimicrococcus*. The microbial diversity obtained in this study obviously differed from those in previous studies at methane seeps in the northern SCS. In June 2013, researchers found that in the Jiaolong methane seep, the majority of the microbial inhabitants at the surface layers (0–6 cm) were *Sulfurimonas, Sulfurovum*, and ANME-1, while SEEP-SRB1, ANME-1, and ANME-2 dominated the deepest layers (8–14 cm). The percentage of ANME-3 was the lowest in all layers, and SEEP-SRB3 and SEEP-SRB4 were not detected (Wu et al., 2018). At the Haiyang 4 hydrate station, only ANME-1 was detected, and the bacterial groups Chloroflexi and JS1 (Atribacteria) were dominant (Zhang et al., 2012). In the GMGS2 gas hydrate station, the majority groups were ANME-1b, ANME-2c, and bacterial group Desulfobacteraceae (Cui et al., 2019). In Haima active methane seep ecosystems in the southwestern SCS, ANME-2a/b was predominant in the upper and middle layers of the SMTZ, whereas ANME-1b outnumbered ANME-2 below the SMTZ, and ANME-3 was absent (Niu et al., 2017).

Methane seeps are island-like habitats, harboring distinct microbial communities (Ruff et al., 2015). The seep communities comprise bacteria and archaea that occur worldwide but are locally selected by the environment. For example, *in situ* temperature, methane concentration (Knittel et al., 2005), oxygen concentration (Meulepas et al., 2009), and sulfate concentration (Yanagawa et al., 2011) can significantly affect the ANME species. A total of >1800 ANME sequences have been reported by far including three types of ANME (ANME-1, -2, and -3) across physiochemically contrasting ecological niches. Among them, ANME-1 and ANME-2 are the most widely distributed in the world and tend to be coupled with syntrophic SEEP-SRB1 bacteria. ANME-1 preferentially grows in hydrogen sulfide-rich and sulfate-depleted environments, while ANME-2 is closely associated with sulfate concentration (Yanagawa et al., 2011) and preferentially grows in sulfate-rich areas. ANME-3 is distributed mainly in methane-seeping mud volcanoes and in some methane seeps (Niemann et al., 2006). Furthermore, in marine sediments, an ecological niche separation occurs where ANME-2a/b dominates the upper layers and ANME-2c and/or ANME-1 outcompetes in deeper zones (Timmers et al., 2017). Our results showed that geochemistry could be the primary force shaping the niche differentiation of functional microbial populations associated with methane-cycling in marine environment.

### Iron-Mediated Anaerobic Oxidation of Methane

The results of incubation experiments (Table 2) showed both SO_4_^2–^-AOM and Fe-AOM occurred in Jiaolong methane seep. Fe-AOM activity appeared below SMTZ at a depth of 8–10 cm, and potential net rates ranged from 20.40 to 37.99 nmol cm^–3^ day^–1^. Compared with net SO_4_^2–^-AOM rates ranging from 10.39 to 900.20 nmol cm ^–3^ day ^–1^, net Fe-AOM rates were 10 times lower.

Our study is the first to discover Fe-AOM in the deep sea methane seeps of the SCS, and potential net rates are higher than those in freshwater and coastal sediments. The potential rate of Fe-AOM was 16.44 nmol cm^–3^ day^–1^ in Eel River Basin (Beal et al., 2009), 3.61 nmol cm^–3^ day^–1^ in brackish coastal sediments (Egger et al., 2015), 3.89 nmol cm^–3^ day^–1^ in coastal Georgia (Segarra et al., 2013), and 3.45 nmol cm^–3^ day^–1^ in deep lake sediment cores (Sivan et al., 2011). The higher rate of Fe-AOM in this study may result from the sufficient supply of iron oxide and massive methane flux. The slope of the northern SCS is one of the world’s most active areas of modern marine sedimentary processes (Huang and Wang, 2007; Luan et al., 2019). A large amount of river-borne terrigenous sediment input leads to exceptionally high amount of iron oxides in the sediments of the northern SCS (Zhang et al., 2007; Liu Z. F. et al., 2016; Liu et al., 2018). Previous research had suggested that different kinds of iron oxides could serve as electron acceptors for Fe-AOM (Bar-Or et al., 2017). In addition, a large amount of unconsumed methane was released into the bottom seawater (22.23 μM, unpublished data), implying a high methane flux in this area. Thus, the combination of these factors probably stimulated the enhanced rate of Fe-AOM processes in the Jiaolong methane seep.

To the best of our knowledge, there is no representative pure culture of Fe-AOM microorganisms from the marine sediment (Liang et al., 2019). But some microorganisms were suspected of being related to metal-AOM in various earlier studies, which suggested that ANME-1, ANME-3 (Beal et al., 2009), ANME-2a, 2c (Scheller et al., 2016), ANME-2d (*Methanoperedens nitroreducens*) (Ettwig et al., 2016; Shen et al., 2019), Candidatus *Methanoperedens ferrireducens* (Cai et al., 2018), or *Methanosarcina acetivorans* (Yan et al., 2018) might be involved in Fe-AOM. Furthermore, it is still possible that other unknown microorganisms perform metal-AOM. Bar-Or et al. (2017) findings highlight the essential role and participation of methanogens archaea and methanotrophic bacteria in the process of Fe-AOM.

In our study, sequencing data showed that the methanotrophic bacteria (such as Candidatus *Methylomirabilis oxyfera*, *Methylobacter, Methylosarcina, Methylomonas*, and *Methylococcus*) were not detected. Under the condition that 20 mM BES was added to inhibit methanogens archaea in the incubation experiments, the Fe-AOM activity appeared below SMTZ. These evidences excluded the potential participation of methanogenic archaea and methanotrophic bacteria in the process of Fe-AOM. CARD-FISH data showed that there was no significant change in the number of ANME at the depth of 6–12 cm, while the number of SRB decreased rapidly, and some ANME-2 and ANME-3 were not coupled with SRB (Figures 6H,I). These results were similar to those from methane seep enrichment samples of the Eel River Basin and Santa Monica Basin, which contained high abundances of ANME-2a and ANME-3 and could decouple the AOM process from SRB activities when metal compounds were added (Beal et al., 2009; Scheller et al., 2016). So we speculated that ANME-2 and ANME-3 were probably involved in Fe-AOM.

Different kinds of IRB were also detected in the Jiaolong seep sediments, such as *Shewanella* (0.01–0.07%), *Geobacter* (0.004–0.02%), *Pseudomonas* (0.01–0.1%), and *Desulfuromonas* (0.11–0.92%). Interestingly, the abundance of IRB was relatively higher in the 12–14 cm depth. To date, two potential ways of Fe-AOM were described in previous studies. (1) ANME oxidizes methane and transfer electrons directly to soluble metal ions or complexes, or solid metal oxides (Ettwig et al., 2016; Scheller et al., 2016); (2) ANME should be partnered with metal-reducing microorganisms to perform metal-AOM, in a way similar to the ANME-SRB consortia (Fu et al., 2016; He et al., 2018). It is worth further exploring whether ANME-2 or ANME-3 should be alone or coupled with IRB to perform Fe-AOM process in the Jiaolong methane seep. As the next step, we would like to use ^14^C-CH_4_ to enrich and cultivate methane-oxidizing microbial populations from the samples. Stable-isotope probing of active AOM would likely provide more hints on AOM metabolisms.

Furthermore, nitrite/nitrate-dependent AOM activity had not been detected in Jiaolong methane seep. Generally, nitrite-dependent AOM is performed by the NC10 bacteria related to Candidatus *Methylomirabilis oxyfera*, and nitrate-dependent AOM is performed by the ANME-2d (Ettwig et al., 2016). In our study, no sequences of Candidatus *Methylomirabilis oxyfera* and ANME-2d were detected. These results suggested that Fe-AOM was probably the dominant non-sulfate AOM pathway in the Jiaolong methane seep.

### Methylotrophic Methanogenesis

Generally, there are three major methanogenic pathways: the acetoclastic, hydrogenotrophic, and methylotrophic pathways (Zhuang et al., 2018). Hydrogenotrophic and acetoclastic methanogenesis are often considered the primary pathways in marine deep sediment (Zhe et al., 2018). Based on thermodynamic laws, SRB and IRB outcompete methanogens for both acetate and hydrogen. Therefore, these methanogens are inhibited during active sulfate reduction and iron reduction (Reeburgh, 2007; Reiche et al., 2010; Zhou et al., 2014; Zhuang et al., 2018). In our study, hydrogenotrophic and acetoclastic methanogenesis were not detected due to active sulfate or iron reducing respiration in the Jiaolong methane seep. However, methylotrophic methanogenesis using non-competitive substrates (methanol) appeared below the 4–6 cm sediment layer, and coexisted with SO_4_^2–^-AOM and Fe-AOM. Most methanogens clustered with the genus *Methanimicrococcus*. It is an obligatory methylotrophic methanogen, that is, it utilizes only non-competitive substrates, such as methanol or methylated compounds (Sprenger et al., 2000; Zeng et al., 2007; Niemann et al., 2009; Wang et al., 2019). Non-competitive substrates, such as methanol, trimethylamine, methylamines, dimethylsulfide, and dimethylsulfoniopropionate are ubiquitous in the marine environment (Oremland et al., 1982).

To date, research on the methylotrophic methanogenesis process has been intensively investigated on non-seep sediments, such as those in the Peruvian Margin (with methanol as the substrate, 0.6–1.95 nmol cm^–3^ d^–1^) (Maltby et al., 2016), Aarhus Bay (with methanol or trimethylamine as the substrate, 0.83–1.11 nmol cm^–3^ d^–1^) (Xiao et al., 2017, 2018), and the Western Mediterranean Sea (with methanol as the substrate, 0.03 nmol cm^–3^ d^–1^) (Zhuang et al., 2018). This process is poorly understood in deep sea methane seep ecosystems due to the technical challenges to discern methane production against the overall high background of net methane consumption in methane seeps ecosystem. To the best of our knowledge, there is only one report in the Sonora Margin cold seep showing methane production by *Methanococcoides burtonii* on non-competitive substrates (with trimethylamine as the substrate, 180∼560 pmol cm^–3^ day^–1^) (Vigneron et al., 2015) in shallow sediments above the SMTZ. The rate of methylotrophic methanogenesis in the Jiaolong methane seep reached a maximum of 5.85 nmol cm^–3^ day^–1^, which was higher than those observed in other areas. Future study is warranted to elucidate the thermal kinetics underlying this microbially mediated process.

In summary, the incubation experiments revealed the coexistence of sulfate-driven AOM, iron-driven AOM, and methylotrophic methanogenesis in Jiaolong methane seep sediments of the northern SCS where terrigenous sediments rich in iron oxide are imported in large quantities. Fe-AOM and methylotrophic methanogenesis are overlooked potential sources and sinks of methane in SCS methane cycle. Globally, large amounts of iron [∼730 Tg/year] from rivers are transported to ocean continental margins (Jickells et al., 2005), and methane seeps are common along continental margins in areas of high primary productivity and tectonic activity. How this methane cycle, which is affected by the large input of iron oxides, will influence the global carbon cycle is worthwhile to study further.

## Data Availability Statement

The datasets generated for this study can be found in the NCBI, PRJNA574743 and PRJNA574745.

## Author Contributions

HL, QY, and HZ designed the research and wrote the manuscript. HL performed the experiments and analyzed the data. All authors commented on the manuscript.

## Conflict of Interest

The authors declare that the research was conducted in the absence of any commercial or financial relationships that could be construed as a potential conflict of interest.

## References

[B1] Bar-Or I. Elvert M. Ecker W. Kushmaro A. Vigderovich H. Zhu Q. Z. (2017). Iron-coupled anaerobic oxidation of methane performed by a mixed bacterial-archaeal community based on poorly reactive minerals. *Environ. Sci. Technol.* 51 12293–12301. 10.1021/acs.est.7b03126 28965392

[B2] Beal E. J. House C. H. Orphan V. J. (2009). Manganese- and iron-dependent marine methane oxidation. *Science* 325 184–187. 10.1126/science.1169984 19589998

[B3] Boetius A. Ravenschlag K. Schubert C. J. Rickert D. Widdel F. Gieseke A. (2000). A marine microbial consortium apparently mediating anaerobic oxidation of methane. *Nature* 407 623–626. 10.1038/35036572 11034209

[B4] Boetius A. Wenzhöfer F. (2013). Seafloor oxygen consumption fuelled by methane from cold seeps. *Nat. Geosci.* 6 725–734. 10.1038/ngeo1926

[B5] Buckley D. H. Baumgartner L. K. Visscher P. T. (2008). Vertical distribution of methane metabolism in microbial mats of the great sippewissett salt marsh. *Environ. Microbiol.* 10 967–977. 10.1111/j.1462-2920.2007.01517.x 18218028

[B6] Cai C. Leu A. O. Xie G. J. Guo J. Feng Y. Zhao J. X. (2018). A methanotrophic archaeon couples anaerobic oxidation of methane to Fe(III) reduction. *ISME Journal* 12 1929–1939. 10.1038/s41396-018-0109-x 29662147 PMC6052012

[B7] Chidthaisong A. Conrad R. (2000). Specificity of chloroform, 2-bromoethanesulfonate and fluoroacetate to inhibit methanogenesis and other anaerobic processes in anoxic rice field soil. *Soil Biology & Biochemistry* 32 977–988. 10.1016/s0038-0717(00)00006-7

[B8] Chronopoulou P. M. Shelley F. Pritchard W. J. Maanoja S. T. Trimmer M. (2017). Origin and fate of methane in the eastern tropical north pacific oxygen minimum zone. *ISME Journal* 11 1386–1399. 10.1038/ismej.2017.6 28244978 PMC5437358

[B9] Cui H. Su X. Chen F. Holland M. Yang S. Liang J. (2019). Microbial diversity of two cold seep systems in gas hydrate-bearing sediments in the South China Sea. *Marine Environmental Ressearch* 144 230–239. 10.1016/j.marenvres.2019.01.009 30732863

[B10] Dekas A. E. Poretsky R. S. Orphan V. J. (2009). Deep-sea archaea fix and share nitrogen in methane-consuming microbial consortia. *Science* 326 422–426. 10.1126/science.1178223 19833965

[B11] Dong D. Li X. (2015). Galatheid and chirostylid crustaceans (Decapoda: Anomura) from a cold seep environment in the northeastern South China Sea. *Zootaxa* 4057 91–105. 26701467 10.11646/zootaxa.4057.1.5

[B12] Egger M. Hagens M. Sapart C. J. Dijkstra N. Van Helmond N. A. G. M. Mogollon J. M. (2017). Iron oxide reduction in methane-rich deep baltic sea sediments. *Geochim. Cosmochim. Acta* 207 256–276. 10.1016/j.gca.2017.03.019

[B13] Egger M. Rasigraf O. Sapart C. J. Jilbert T. Jetten M. S. M. Rockmann T. (2015). Iron-mediated anaerobic oxidation of methane in brackish coastal sediments. *Environ. Sci. Technol.* 49 277–283. 10.1021/es503663z 25412274

[B14] Eickhorst T. Tippkötter R. (2008). Improved detection of soil microorganisms using fluorescence in situ hybridization (FISH) and catalyzed reporter deposition (CARD-FISH). *Soil Biol. Biochem.* 40 1883–1891. 10.1016/j.soilbio.2008.03.024

[B15] Ettwig K. F. Zhu B. Speth D. Keltjens J. T. Jetten M. S. Kartal B. (2016). Archaea catalyze iron-dependent anaerobic oxidation of methane. *Proc. Natl. Acad. Sci. U.S.A.* 113 12792–12796. 10.1073/pnas.1609534113 27791118 PMC5111651

[B16] Expedition 349 Scientists (2014). South China Sea tectonics: opening of the South China Sea and its implications for southeast Asian tectonics, climates, and deep mantle processes since the late Mesozoic. *Int. Ocean Discov. Progr. Preliminary Rep.* 349, 1–109.

[B17] Feng D. Qiu J.-W. Hu Y. Peckmann J. Guan H. Tong H. (2018). Cold seep systems in the South China Sea: an overview. *J. Asian Earth Sci.* 168 3–16. 10.1016/j.jseaes.2018.09.021

[B18] Fu L. Li S. W. Ding Z. W. Ding J. Lu Y. Z. Zeng R. J. (2016). Iron reduction in the DAMO/Shewanella oneidensis MR-1 coculture system and the fate of Fe(II). *Water Res.* 88 808–815. 10.1016/j.watres.2015.11.011 26599434

[B19] Gong L. Li X. Qiu J. W. (2015). Two new species of Hexactinellida (Porifera) from the South China Sea. *Zootaxa* 4034 182–192. 26624437 10.11646/zootaxa.4034.1.9

[B20] Guan H. X. Birgel D. Peckmann J. Liang Q. Y. Feng D. Yang S. X. (2018). Lipid biomarker patterns of authigenic carbonates reveal fluid composition and seepage intensity at Haima cold seeps. South China Sea. *J. Asian Earth Sci.* 168 163–172. 10.1016/j.jseaes.2018.04.035

[B21] Guan H. X. Feng D. Wu N. Y. Chen D. F. (2016). Methane seepage intensities traced by biomarker patterns in authigenic carbonates from the South China Sea. *Organic Geochem.* 91 109–119. 10.1016/j.orggeochem.2015.11.007

[B22] Haroon M. F. Hu S. Shi Y. Imelfort M. Keller J. Hugenholtz P. (2013). Anaerobic oxidation of methane coupled to nitrate reduction in a novel archaeal lineage. *Nature* 500 567–570. 10.1038/nature12375 23892779

[B23] He Z. Zhang Q. Feng Y. Luo H. Pan X. Gadd G. M. (2018). Microbiological and environmental significance of metal-dependent anaerobic oxidation of methane. *Sci. Total Environ.* 61 759–768. 10.1016/j.scitotenv.2017.08.140 28830047

[B24] Huang W. Wang P. (2007). Statistics of sediment mass in the South China Sea: method and results. *Front. Earth Sci. China* 1:88–96. 10.1007/s11707-007-0012-7

[B25] Jiang H. Dong H. Ji S. Ye Y. Wu N. (2007). Microbial diversity in the deep marine sediments from the Qiongdongnan Basin in South China Sea. *Geomicrobiology Journal* 24 505–517. 10.1080/01490450701572473

[B26] Jickells T. D. An Z. S. Andersen K. K. Baker A. R. Bergametti G. Brooks N. (2005). Global iron connections between desert dust, ocean biogeochemistry, and climate. *Science* 308 67–71. 10.1126/science.1105959 15802595

[B27] Kleindienst S. Ramette A. Amann R. Knittel K. (2012). Distribution and in situ abundance of sulfate-reducing bacteria in diverse marine hydrocarbon seep sediments. *Environ. Microbiol.* 14 2689–2710. 10.1111/j.1462-2920.2012.02832.x 22882476

[B28] Knittel K. Boetius A. (2009). Anaerobic oxidation of methane: progress with an unknown process. *Ann. Rev. Microbiol.* 63 311–334. 10.1146/annurev.micro.61.080706.093130 19575572

[B29] Knittel K. Losekann T. Boetius A. Kort R. Amann R. (2005). Diversity and distribution of methanotrophic archaea at cold seeps. *Appl. Environ. Microbiol.* 71 467–479. 10.1128/aem.71.1.467-479.2005 15640223 PMC544223

[B30] Könneke M. Bernhard A. E. José R. Walker C. B. Waterbury J. B. Stahl D. A. (2005). Isolation of an autotrophic ammonia-oxidizing marine archaeon. *Nature* 437 543–546. 10.1038/nature03911 16177789

[B31] Krüger M. Treude T. Wolters H. Nauhaus K. Boetius A. (2005). Microbial methane turnover in different marine habitats. *Palaeogeogr. Palaeoclimatol. Palaeoecol.* 227 6–17. 10.1016/j.palaeo.2005.04.031

[B32] Liang L. Wang Y. Sivan O. Wang F. (2019). Metal-dependent anaerobic methane oxidation in marine sediment: insights from marine settings and other systems. *Sci. China Life Sci.* 62 1287–1295. 10.1007/s11427-018-9554-5 31209798

[B33] Liu C. Li H. Zhang Y. Si D. Chen Q. (2016). Evolution of microbial community along with increasing solid concentration during high-solids anaerobic digestion of sewage sludge. *Bioresour. Technolol.* 216 87–94. 10.1016/j.biortech.2016.05.048 27235970

[B34] Liu Z. F. Zhao Y. L. Colin C. Stattegger K. Wiesner M. G. Huh C. A. (2016). Source-to-sink transport processes of fluvial sediments in the South China Sea. *Earth Sci. Rev.* 153 238–273. 10.1016/j.earscirev.2015.08.005

[B35] Liu J. R. Izon G. Wang J. S. Antler G. Wang Z. Zhao J. (2018). Vivianite formation in methane-rich deep-sea sediments from the South China Sea. *Biogeosciences* 15 6329–6348. 10.5194/bg-15-6329-2018

[B36] Luan X. Ran W. Wang K. Wei X. Shi Y. Zhang H. (2019). New interpretation for the main sediment source of the rapidly deposited sediment drifts on the northern slope of the South China Sea. *J. Asian Earth Sci.* 171 118–133. 10.1016/j.jseaes.2018.11.004

[B37] Maltby J. Sommer S. Dale A. W. Treude T. (2016). Microbial methanogenesis in the sulfate-reducing zone of surface sediments traversing the Peruvian margin. *Biogeosciences* 13 283–299. 10.5194/bg-13-283-2016

[B38] Manz W. Amann R. Ludwig W. Wagner M. Schleifer K. H. (1992). Phylogenetic oligodeoxynucleotide probes for the major subclasses of *proteobacteria* - problems and solutions. *Syst. Appl. Microbiol.* 15 593–600. 10.1016/s0723-2020(11)80121-9

[B39] Meulepas R. J. W. Jagersma C. G. Gieteling J. Buisman C. J. N. Stams A. J. M. Lens P. N. L. (2009). Enrichment of anaerobic methanotrophs in sulfate-reducing membrane bioreactors. *Biotechnol. Bioeng.* 104 458–470. 10.1002/bit.22412 19544305

[B40] Nauhaus K. Treude T. Boetius A. Kruger M. (2005). Environmental regulation of the anaerobic oxidation of methane: a comparison of ANME-I and ANME-II communities. *Environ. Microbiol.* 7 98–106. 10.1111/j.1462-2920.2004.00669.x 15643940

[B41] Niemann H. Fischer D. Graffe D. Knittel K. Montiel A. Heilmeyer O. (2009). Biogeochemistry of a low-activity cold seep in the Larsen B area, western Weddell Sea, Antarctica. *Biogeosciences* 6 2383–2395. 10.5194/bg-6-2383-2009

[B42] Niemann H. Lösekann T. De Beer D. Elvert M. Nadalig T. Knittel K. (2006). Novel microbial communities of the Haakon Mosby mud volcano and their role as a methane sink. *Nature* 443 854–858. 10.1038/nature05227 17051217

[B43] Niu M. Fan X. Zhuang G. Liang Q. Wang F. (2017). Methane-metabolizing microbial communities in sediments of the Haima cold seep area, northwest slope of the South China Sea. *FEMS Microbiol. Ecol.* 93:fix101. 10.1093/femsec/fix101 28934399

[B44] Oda Y. Sakamoto M. Iwasaki Y. Nagasaka S. Ha J.-Y. Chang K.-H. (2019). Inter-clonal variation in copper sensitivity in *Bosmina longirostris* with different exposure histories. *Water Air Soil Pollut.* 230:109.

[B45] Offre P. Spang A. Schleper C. (2013). Archaea in biogeochemical cycles. *Ann. Rev. Microbiol.* 67 437–457. 10.1146/annurev-micro-092412-155614 23808334

[B46] Oni O. Miyatake T. Kasten S. Richter-Heitmann T. Fischer D. Wagenknecht L. (2015). Distinct microbial populations are tightly linked to the profile of dissolved iron in the methanic sediments of the Helgoland mud area. North Sea. *Front. Microbiol.* 6:365. 10.3389/fmicb.2015.00365 25983723 PMC4416451

[B47] Orcutt B. N. Joye S. B. Kleindienst S. Knittel K. Ramette A. Reitz A. (2010). Impact of natural oil and higher hydrocarbons on microbial diversity, distribution, and activity in Gulf of Mexico cold-seep sediments. *Deep Sea Res. Part II Top. Stud Oceanogr.* 57 2008–2021. 10.1016/j.dsr2.2010.05.014

[B48] Oremland R. S. Capone D. G. (1988). Use of “Specific” inhibitors in biogeochemistry and microbial ecology. *Adv. Microb. Ecol.* 10 285–383. 10.1007/978-1-4684-5409-3_8

[B49] Oremland R. S. Marsh L. M. Polcin S. (1982). Methane production and simultaneous sulfate reduction in anoxic, salt-marsh sediments. *Nature* 296 143–145. 10.1038/296143a0

[B50] Paull C. K. Hecker B. Commeau R. Freeman-Lynde R. P. Neumann C. Corso W. P. (1984). Biological communities at the Florida escarpment resemble hydrothermal vent taxa. *Science* 226 965–967. 10.1126/science.226.4677.965 17737352

[B51] Pernthaler A. Pernthaler J. Amann R. (2002). Fluorescence in situ hybridization and catalyzed reporter deposition for the identification of marine bacteria. *Appl. Environ. Microbiol.* 68 3094–3101. 10.1128/aem.68.6.3094-3101.2002 12039771 PMC123953

[B52] Raghoebarsing A. A. Pol A. Van De Pas-Schoonen K. T. Smolders A. J. Ettwig K. F. Rijpstra W. I. (2006). A microbial consortium couples anaerobic methane oxidation to denitrification. *Nature* 440 918–921. 10.1038/nature04617 16612380

[B53] Reeburgh W. S. (2007). Oceanic methane biogeochemistry. *Chem. Rev.* 38 486–513. 10.1021/cr050362v 17261072

[B54] Reiche M. Torburg G. Küsel K. (2010). Competition of Fe(III) reduction and methanogenesis in an acidic fen. *FEMS Microbiol. Ecol.* 65 88–101. 10.1111/j.1574-6941.2008.00523.x 18559015

[B55] Riedinger N. Formolo M. J. Lyons T. W. Henkel S. Beck A. Kasten S. (2014). An inorganic geochemical argument for coupled anaerobic oxidation of methane and iron reduction in marine sediments. *Geobiology* 12 172–181. 10.1111/gbi.12077 24460948

[B56] Ruff S. E. Biddle J. F. Teske A. P. Knittel K. Boetius A. Ramette A. (2015). Global dispersion and local diversification of the methane seep microbiome. *Proc. Natl. Acad. Sci. U.S.A.* 112 4015–4020. 10.1073/pnas.1421865112 25775520 PMC4386351

[B57] Scheller S. Yu H. Chadwick G. L. Mcglynn S. E. Orphan V. J. (2016). Artificial electron acceptors decouple archaeal methane oxidation from sulfate reduction. *Science* 351 703–707. 26912857 10.1126/science.aad7154

[B58] Schreiber L. Holler T. Knittel K. Meyerdierks A. Amann R. (2010). Identification of the dominant sulfate-reducing bacterial partner of anaerobic methanotrophs of the ANME-2 clade. *Environ. Microbiol.* 12 2327–2340. 21966923 10.1111/j.1462-2920.2010.02275.x

[B59] Seeberg-Elverfeldt J. Schluter M. Feseker T. Kolling M. (2005). Rhizon sampling of porewaters near the sediment-water interface of aquatic systems. *Limnol. Oceanogr. Methods* 3 361–371. 10.4319/lom.2005.3.361

[B60] Segarra K. E. Schubotz F. Samarkin V. Yoshinaga M. Y. Hinrichs K. U. Joye S. B. (2015). High rates of anaerobic methane oxidation in freshwater wetlands reduce potential atmospheric methane emissions. *Nat. Commun.* 6:7477. 10.1038/ncomms8477 26123199

[B61] Segarra K. E. A. Comerford C. Slaughter J. Joye S. B. (2013). Impact of electron acceptor availability on the anaerobic oxidation of methane in coastal freshwater and brackish wetland sediments. *Geochim. Cosmochim. Acta* 115 15–30. 10.1016/j.gca.2013.03.029

[B62] Sela-Adler M. Ronen Z. Herut B. Antler G. Vigderovich H. Eckert W. (2017). Co-existence of methanogenesis and sulfate reduction with common substrates in sulfate-rich estuarine sediments. *Front. Microbiol.* 8:766. 10.3389/fmicb.2017.00766 28529500 PMC5418336

[B63] Shen L. D. Ouyang L. Zhu Y. Trimmer M. (2019). Active pathways of anaerobic methane oxidation across contrasting riverbeds. *ISME J.* 13 752–766. 10.1038/s41396-018-0302-y 30375505 PMC6461903

[B64] Sivan O. Adler M. Pearson A. Gelman F. Bar-Or I. John S. G. (2011). Geochemical evidence for iron-mediated anaerobic oxidation of methane. *Limnol. Oceanogr.* 56 1536–1544. 10.4319/lo.2011.56.4.1536

[B65] Sivan O. Antler G. Turchyn A. V. Marlow J. J. Orphan V. J. (2014). Iron oxides stimulate sulfate-driven anaerobic methane oxidation in seeps. *Proc. Natl. Acad. Sci. U.S.A.* 111 4139–4147. 25246590 10.1073/pnas.1412269111 PMC4209987

[B66] Sprenger W. W. Van Belzen M. C. Rosenberg J. Hackstein J. H. Keltjens J. T. (2000). *Methanomicrococcus blatticola* gen. nov., sp. nov., a methanol- and methylamine-reducing methanogen from the hindgut of the cockroach *Periplaneta americana*. *Int. J. Syst. Evol. Microbiol.* 6 1989–1999. 10.1099/00207713-50-6-1989 11155972

[B67] Timmers P. H. Welte C. U. Koehorst J. J. Plugge C. M. Jetten M. S. Stams A. J. (2017). Reverse methanogenesis and respiration in methanotrophic archaea. *Archaea* 2017:1654237. 10.1155/2017/1654237 PMC5244752 28154498

[B68] Treude T. Krause S. Maltby J. Dale A. W. Coffin R. Hamdan L. J. (2014). Sulfate reduction and methane oxidation activity below the sulfate-methane transition zone in Alaskan Beaufort Sea continental margin sediments: implications for deep sulfur cycling. *Geochim.Cosmochim. Acta* 144 217–237. 10.1016/j.gca.2014.08.018

[B69] Treude T. Niggemann J. Kallmeyer J. Wintersteller P. Schubert C. J. Boetius A. (2005). Anaerobic oxidation of methane and sulfate reduction along the Chilean continental margin. *Geochim.Cosmochim. Acta* 69 2767–2779. 10.1016/j.gca.2005.01.002

[B70] Valentine D. L. (2011). Emerging topics in marine methane biogeochemistry. *Ann. Rev. Mar. Sci.* 3 147–171. 10.1146/annurev-marine-120709-142734 21329202

[B71] Vigderovich H. Liang L. Herut B. Wang F. Wurgaft E. Rubin-Blum M. (2019). Evidence for microbial iron reduction in the methanic sediments of the oligotrophic southeastern Mediterranean continental shelf. *Biogeosciences* 16 3165–3181. 10.5194/bg-16-3165-2019

[B72] Vigneron A. L’haridon S. Godfroy A. Roussel E. G. Cragg B. A. Parkes R. J. (2015). Evidence of active methanogen communities in shallow sediments of the Sonora Margin cold seeps. *Appl. and Environ. Microbiol.* 81 3451–3459. 10.1128/aem.00147-15 25769831 PMC4407212

[B73] Wallner G. Amann R. Beisker W. (1993). Optimizing fluorescent in situ hybridization with rRNA-targeted oligonucleotide probes for flow cytometric identification of microorganisms. *Cytometry* 14 136–143. 10.1002/cyto.990140205 7679962

[B74] Wang B. Liu F. Zheng S. Hao Q. (2019). Trophic strategy of diverse methanogens across a river-to-sea gradient. *J. Microbiol.* 57 470–478. 10.1007/s12275-019-8482-3 31054138

[B75] Wu Y. Qiu J. W. Qian P. Y. Wang Y. (2018). The vertical distribution of prokaryotes in the surface sediment of Jiaolong cold seep at the northern South China Sea. *Extremophiles* 22 1–12. 29442249 10.1007/s00792-018-1012-0

[B76] Xiao K. Q. Beulig F. Kjeldsen K. U. Jørgensen B. B. Risgaard-Petersen N. (2017). Concurrent methane production and oxidation in surface sediment from Aarhus Bay, Denmark. *Front. Microbiol.* 8:1198. 10.3389/fmicb.2017.01198 28713339 PMC5492102

[B77] Xiao K. Q. Beulig F. Røy H. Jørgensen B. B. Risgaard-Petersen N. (2018). Methylotrophic methanogenesis fuels cryptic methane cycling in marine surface sediment. *Limno. Oceanogr.* 63 1519–1527. 10.1002/lno.10788

[B78] Xu N. Tan G. C. Wang H. Y. Gai X. P. (2016). Effect of biochar additions to soil on nitrogen leaching, microbial biomass and bacterial community structure. *Eur. J. Soil Biol.* 74 1–8. 10.1016/j.ejsobi.2016.02.004

[B79] Yan Z. Joshi P. Gorski C. A. Ferry J. G. (2018). A biochemical framework for anaerobic oxidation of methane driven by Fe(III)-dependent respiration. *Nat. Commun.* 9:1642. 10.1038/s41467-018-04097-9 PMC5915437 29691409

[B80] Yanagawa K. Sunamura M. Lever M. A. Morono Y. Hiruta A. Ishizaki O. (2011). Niche separation of methanotrophic archaea (ANME-1 and-2) in methane-seep sediments of the eastern japan sea offshore joetsu. *Geomicrobiol. J.* 28 118–129. 10.1080/01490451003709334

[B81] Zeng Y. Li H. Jiao N. (2007). Phylogenetic diversity of planktonic archaea in the estuarine region of East China Sea. *Microbiol. Res.* 162 26–36. 10.1016/j.micres.2006.03.007 16914298

[B82] Zhang Y. Su X. Chen F. Wang Y. Y. Jiao L. Dong H. L. (2012). Microbial diversity in cold seep sediments from the northern South China Sea. *Geosci. Front.* 3 301–316. 10.1016/j.gsf.2011.11.014

[B83] Zhang Y. G. Ji J. Balsam W. L. Liu L. Chen J. (2007). High resolution hematite and goethite records from ODP 1143, South China Sea: co-evolution of monsoonal precipitation and El Niño over the past 600,000 years. *Earth Planet. Sci. Lett.* 264 136–150. 10.1016/j.epsl.2007.09.022

[B84] Zhe L. Shao N. Akinyemi T. Whitman W. B. (2018). Methanogenesis. *Curr. Biol.* 28 727–732. 10.1016/j.cub.2018.05.021 29990451

[B85] Zhou J. Bruns M. A. Tiedje J. M. (1996). DNA recovery from soils of diverse composition. *Appl. Environ. Microbiol.* 62 316–322. 10.1128/aem.62.2.316-322.1996 8593035 PMC167800

[B86] Zhou S. Xu J. Yang G. Zhuang L. (2014). Methanogenesis affected by the co-occurrence of iron(III) oxides and humic substances. *FEMS Microbiol. Ecol.* 88 107–120. 10.1111/1574-6941.12274 24372096

[B87] Zhuang G. C. Elling F. J. Nigro L. M. Samarkin V. Joye S. B. Teske A. (2016). Multiple evidence for methylotrophic methanogenesis as the dominant methanogenic pathway in hypersaline sediments from the Orca Basin. Gulf of Mexico. *Geochim. Cosmochim. Acta* 187 1–20. 10.1016/j.gca.2016.05.005

[B88] Zhuang G. C. Heuer V. B. Lazar C. S. Goldhammer T. Wendt J. Samarkin V. A. (2018). Relative importance of methylotrophic methanogenesis in sediments of the Western Mediterranean Sea. *Geochim.Cosmochim. Acta* 224 171–186. 10.1016/j.gca.2017.12.024

