# Supplementary material for: Niche Differentiation of Sulfate- and Iron-Dependent Anaerobic Methane Oxidation and Methylotrophic Methanogenesis in Deep Sea Methane Seeps
Source: Front Microbiol. 2020 Jul 8;11:1409. doi: 10.3389/fmicb.2020.01409 (PMC7360803; doi:10.3389/fmicb.2020.01409)
Supplement: Supplementary file 1 [file Table_1.DOCX]

Supplementary Material

## Supplementary Figures

A
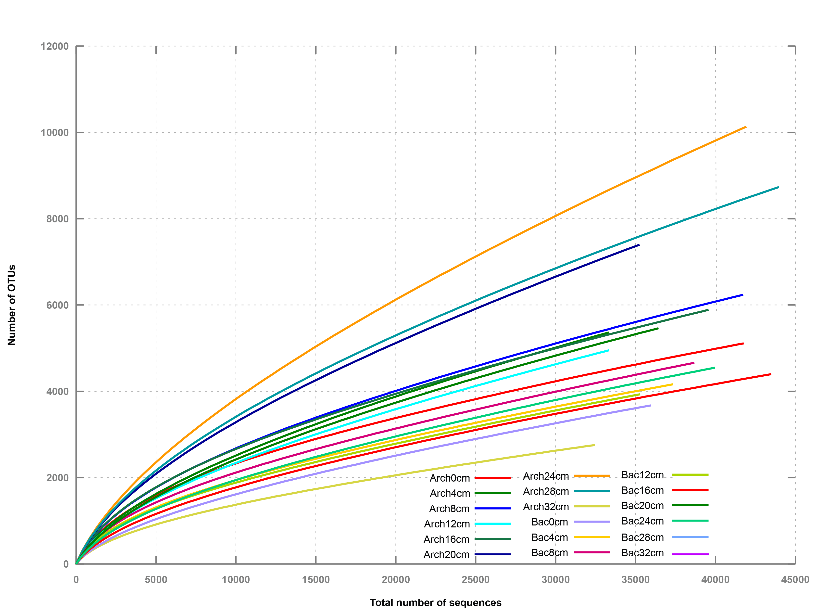


B
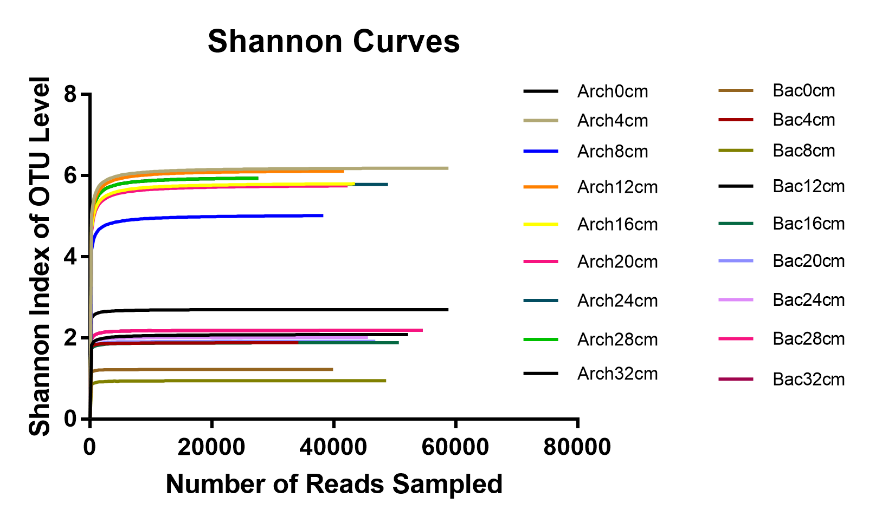


**Supplementary Figure S1.** OTU-level rarefaction (observed species) (A) and Shannon diversity index curves (B) of sediments. Colored curves represent each sample. Not all the rarefaction curves plateaued at the current sequencing depth, but the Shannon index rarefaction curves reached stable values, indicating that most of the microorganism had already been detected. The other uncaptured rare species would not affect our conclusions.


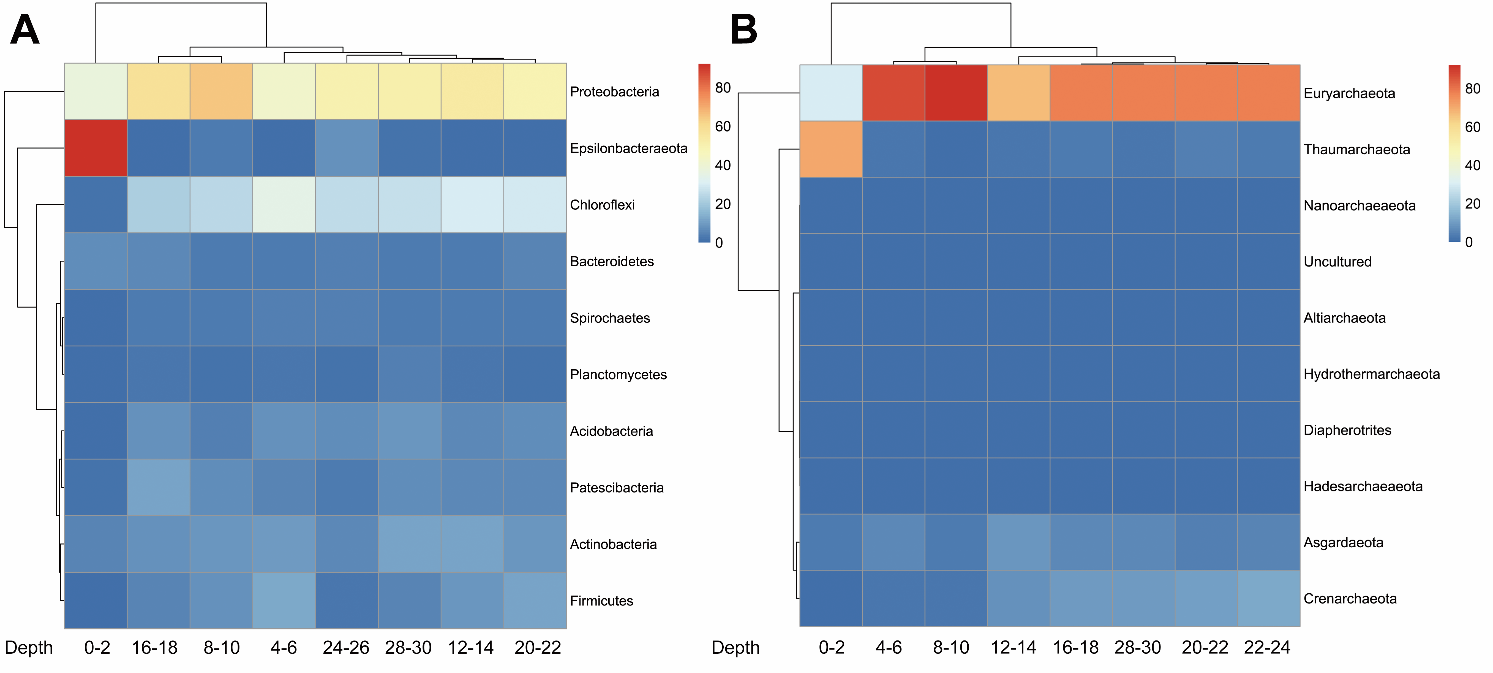


Figure S2 16S rRNA genes sequences abundance at top 10 phylum level obtained from different depths sediments. A, Bacteria; B, Archaea.

The bacterial sequences mainly belonged to the following phyla: Epsilonbacteraeota, Proteobacteria, Chloroflexi, Actinobacteria and Firmicutes (Figure 3A). Epsilonbacteraeota was the most abundant phylum in surface sediments (61.9%). Proteobacteria was the most abundant phylum at the depths of 4–30 cm, with abundance ranging from 25.0% to 43.8634%, and the highest abundance of Proteobacteria was found at the depth of 8–10 cm. Chloroflexi was also highly abundant at the depths of 4–28 cm, ranging from 0.78% to 23.01%, and the highest abundance of Chloroflexi was found at 4–6 cm. PCoA of archaea from all samples showed differences between sediments at depths of 0–6 cm and lower sediments (Figure S2B). The identified archaeal sequences mainly belonged to the following phyla: Euryarchaeota, Thaumarchaeota and Crenarchaeota (Figure 3B). Thaumarchaeota was the most abundant phylum in surface sediments with an abundance of 70.65%. The abundance of Euryarchaeota was 29.84% in surface sediments, but it was found to be more dominant in deep sediments (4–30cm) with abundance ranging from 66.85% to 92.23%. The abundance of Crenarchaeota increased with depth, ranging from 0.09% to 10.87%.


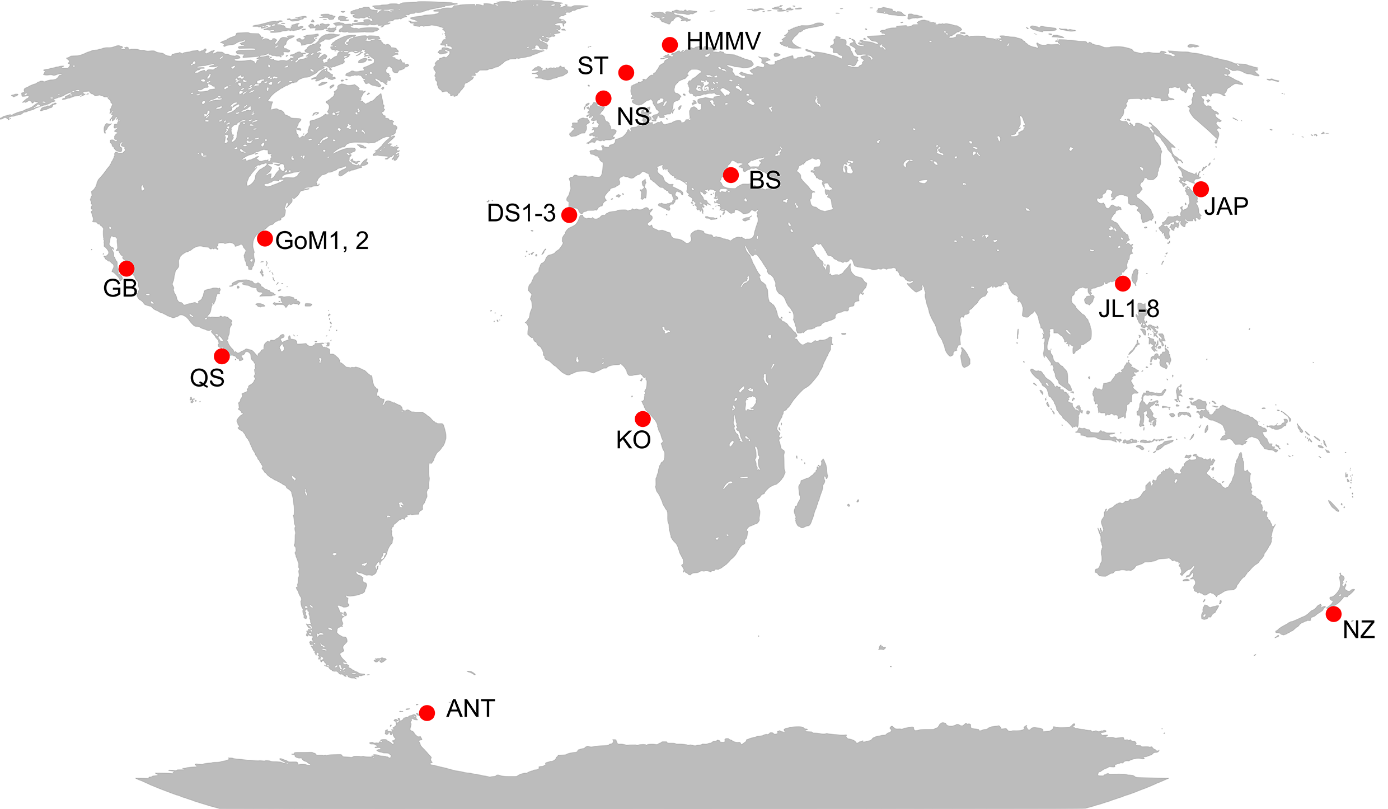


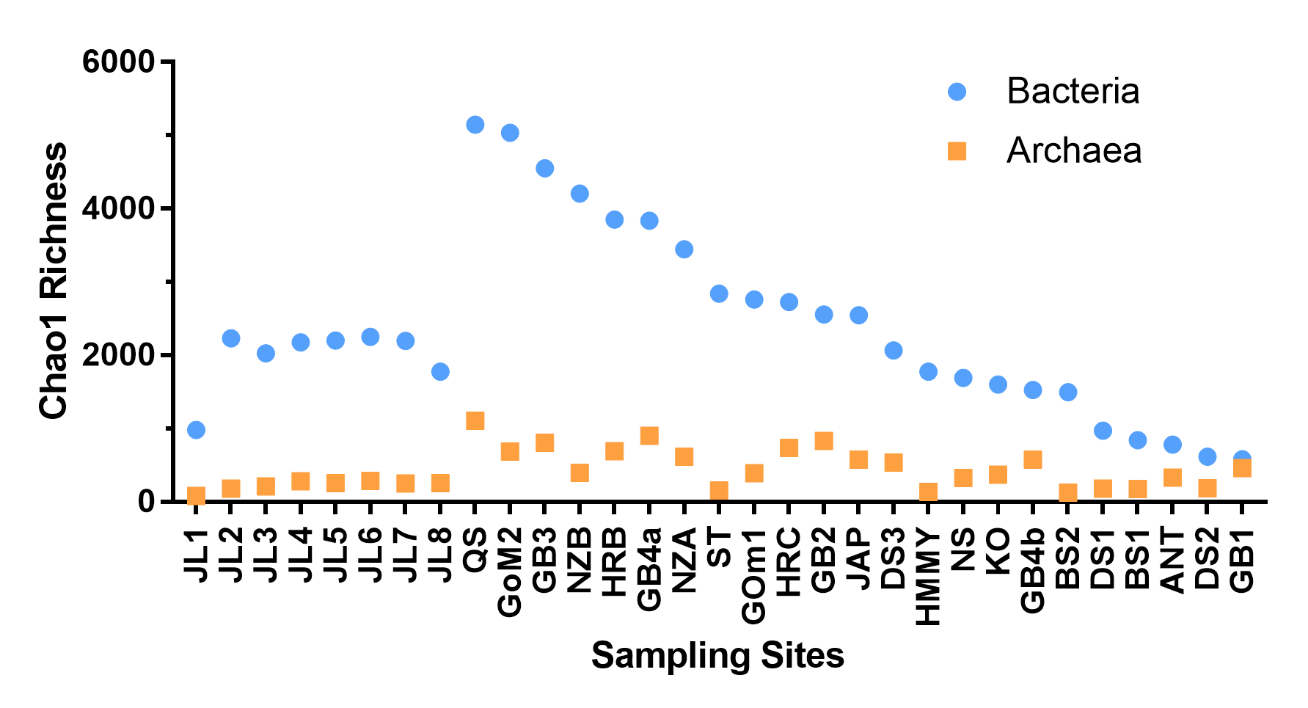


Figure S3 Richness estimates based on archaeal and bacterial OTU 0.03. Revised from Ruff et al. (Ruff et al., 2015). Datasets with spatially distributed sites are numbered (e.g., DS1-10). ANT, Low-activity Antarctic seep; BS, Black Sea microbial reef; DS, Gulf of Cadiz mud volcanoes; GB, Guaymas Basin hot seeps; GoM, Gulf of Mexico seeps; HMMV, Håkon Mosby mud volcano; HR, Hydrate Ridge seeps; JAP, Japanese Trench seep; KO, Congo Basin (REGAB) seep; NS, North Sea seep; NZ, New Zealand seeps; QS, Quepos Slide seep (Costa Rica); and ST, Storegga Slide seep (Norway). Across all seep ecosystems, archaeal diversity was lower than bacterial diversity.

Table S1 Incubation experiments

BES (20 mM): 2-bromoethanosulfonate, methanogenesis inhibitor; Molybdate (20mM): inhibitor of SO_4_^2-^-AOM.

| Treatments | | Depth (cm) | | | | | | | |
| --- | --- | --- | --- | --- | --- | --- | --- | --- | --- |
|  |  | 0-2 | 4-6 | 8-10 | 12-14 | 16-18 | 20-22 | 24-26 | 28-30 |
| AOM  (nmol g^-1^d^-1^) | (1) Without any addition | 0.210  0.304 | 1.250  1.264 | 1.898  2.196 | 1.135  1.381 | 0.610  0.640 | 0.105  0.135 | 0.145  0.155 | 0.092  0.110 |
|  | (2) Sulfate (5 mM) | 10.450  10.850 | 410.540  391.868 | 910.950  893.546 | 810.550  792.598 | 770.420  790.036 | 756.026  744.262 | 620.038  581.338 | 590.450  571.330 |
|  | (3) BES + Molybdate | - | - | 0.028  0.036 | 0.021  0.029 | 0.027  0.035 | 0.033  0.037 | 0.021  0.031 | 0.031  0.039 |
|  | (4) BES + Molybdate + Ferrihydrite (10 mM) | - | - | 30.087  29.981 | 38.220  37.818 | 20.031  20.835 | 34.856  35.254 | 34.925  35.121 | 30.334  29.734 |
|  | (5) BES + Molybdate + Nitrite (5 mM) | - | - | 0.026  0.036 | 0.029  0.021 | 0.044  0.022 | 0.036  0.030 | 0.018  0.032 | 0.030  0.032 |
|  | (6 BES + Molybdate + Nitrate (10 mM) | - | - | 0.029  0.031 | 0.023  0.017 | 0.033  0.025 | 0.038  0.024 | 0.025  0.023 | 0.039  0.035 |
| Methanogenesis  (nmol g^-1^d^-1^) | (1) Molybdate | - | 0.022  0.024 | 0.092  0.074 | 0.085  0.079 | 0.162  0.182 | 0.114  0.150 | 0.142  0.102 | 0.094  0.114 |
|  | (2) Molybdate + Methanol (20 mM) | - | 0.044  0.040 | 2.160  2.004 | 3.085  3.075 | 6.038  6.004 | 6.044  5.822 | 5.305  5.543 | 5.005  5.205 |
|  | (3) Molybdate + H_2_/CO_2_ (80/20%) | - | 0.011  0.013 | 0.091  0.075 | 0.088  0.094 | 0.174  0.154 | 0.144  0.122 | 0.124  0.122 | 0.124  0.106 |
|  | (4) Molybdate + Acetate (20 mM) | - | 0.016  0.024 | 0.074  0.076 | 0.092  0.076 | 0.162  0.184 | 0.149  0.155 | 0.104  0.116 | 0.094  0.086 |
